# Supplementary material for: Unifying description of competing orders in two-dimensional quantum magnets
Source: Nat Commun. 2019 Sep 18;10:4254. doi: 10.1038/s41467-019-11727-3 (PMC6751208; doi:10.1038/s41467-019-11727-3)
Supplement: Supplementary file 1 — Supplementary Information [file 41467_2019_11727_MOESM1_ESM.pdf]

Supplementary materials for Unifying Description of Competing Orders in Two Dimensional Quantum Magnets

Xue-Yang Song,[1] Chong Wang,[2,1] Ashvin Vishwanath,[1,*] and Yin-Chen He[2,1]

[1] Department of Physics, Harvard University,Cambridge MA 02138, USA
[2] Perimeter Institute for Theoretical Physics, Waterloo, ON N2L 2Y5, Canada

(Dated: July 22, 2019)

Supplementary Note 1. MEAN-FIELD ANSATZ, PROJECTIVE SYMMETRY GROUP AND ALGEBRAIC RELATIONS

A. Square

We adopt a mean-field where $t_{ij} = (-1)^y$ for horizontal links and $t_{ij} = 1$ for vertical links on square lattice which creates π flux on every plaquette. This choice enlarges the unit cell to contain two sites (sublattice A, B) with a vertical link. There're two gapless points in the reduced Brillouin zone at $\mathbf{Q} = (\pi/\mathbf{2}, \pi), \mathbf{Q'} = -\mathbf{q}$.

The projective symmetry group reads (for staggered flux state)

$$
\begin{aligned}
&T_1 : \psi \to i\mu^3\sigma^2\tau^3\psi^* \quad T_2 : \psi \to i\mu^3\sigma^2\tau^1\psi^* \\
&R_x : \psi \to \tau^3\mu^3\psi \quad C_4 : \psi \to \frac{1}{\sqrt{2}}\mu^3\sigma^2(I - i\tau^2)e^{i\frac{\pi}{4}\mu^1}\psi^* \\
&\mathcal{T} : \psi \to \tau^2\mu^1\psi^*. \qquad (1)\\
&\mathcal{C} : \psi \to i\mu^3\sigma^2\psi^* \qquad (2)
\end{aligned}
$$

where $\mathcal{C}$ denotes charge conjugation that reverses the flux $\phi \to -\phi$.

The six monopoles transform as the six fermion bilinears $\bar\psi\tau^{1/2/3}\psi, \bar\psi\sigma^{1/2/3}\psi$ [1] with an additional Berry phase (see also Supplementary Note 2) (up to $\mathcal{T}, \mathcal{C}, \mathcal{R}$ which change the monopole charge). There are algebraic relations between the symmetry group which could help restrict the choice of Berry phase for monopoles in the π-flux phase (for $T_{1/2} : \theta_{1/2}, C_4 : \theta_C$, which read:

$$
\begin{aligned}
&T_1T_2 = T_2T_1 \quad [\mathcal{T}, T_{1/2}] = 0 \quad [\mathcal{T}, R] = 0 \\
&C_4T_1 = T_2C_4 \quad C_4T_2 = (T_1)^{-1}C_4 \quad T_1R = RT_1 \quad T_2RT_2 = R \quad (C_4)^4 = R^2 = 1 \qquad (3)
\end{aligned}
$$

which dictates

$$\theta_1 = \theta_2 = 0, \pi \quad \theta_C = n\pi/2, n \in \mathbb{Z}. \qquad (4)$$

Numerically we find $\theta_1 = \theta_2 = \pi$.

B. Honeycomb

On honeycomb lattice, with mean-field ansatz of uniform fermion hopping, one could similarly work out the PSG and the constraints on monopole quantum numbers. The Dirac points stay at momenta $\mathbf{Q} = (\frac{2\pi}{3}, \frac{2\pi}{3}), \mathbf{Q'} = -\mathbf{Q}$. The physical symmetries act as

* To whom correspondence should be addressed, e-mail: avishwanath@g.harvard.edu.

$$T_{1/2}:\psi\to e^{-i\frac{2\pi}{3}\tau^3}\psi$$
$$C_6:\psi\to -ie^{-i\frac{\pi}{6}\mu^3}\tau^1 e^{-i\frac{2\pi}{3}\tau^3}\psi \quad R:\psi\to-\mu^2\tau^2\psi$$
$$\mathcal{T}:\psi\to -i\sigma^2\mu^2\tau^2\psi \quad \text{charge conjugation } C:\psi\to\mu^1\psi^\dagger \tag{5}$$

where $T_{1/2}$ is the translation along two basis vectors with $2\pi/3$ angle between them,C_6 is $\pi/3$ rotation around a center of a honeycomb plaquette, and R denotes reflection along the direction of the unit cell.

From the transformation of Dirac fermions, one gets transformation of fermion masses.

The algebraic relations between symmetries constrain the Berry phase of the Dirac sea as follows: (berry phase for translation $T_{1/2}:\theta_{1/2}$, for $C_6:\theta_C$)

$$\mathcal{T}^2=1 \quad [U,\mathcal{T}]=0 \quad C_6^6=1:6\theta_C=0(\mod 2\pi)$$
$$C_3T_2=T_1C_3:\theta_2=\theta_1 \quad T_1T_2C_3T_1=C_3:2\theta_1+\theta_2=0(\mod 2\pi)$$
$$T_2C_6T_1=C_6:\theta_1+\theta_2=0(\mod 2\pi) \tag{6}$$

where $C_3=RC_6^{-1}R^{-1}C_6$ is the 3-fold rotation around a A sub-lattice site. Together they stipulate that

$$\theta_1=\theta_2=0 \quad \theta_C=n\pi/3. \tag{7}$$

Note it's the relations involving translation and rotation such as $C_3T_2=T_1C_3$ that enforces the vanishing berry phase under translations.

C. Triangular lattice

There's a "staggered π flux" configuration of t_{ij} on the triangular lattice. We choose a particular gauge of t_{ij} to realize this mean field as in Supplementary Fig 1. Under appropriate basis the low-energy Hamiltonian reads as the standard form with 4 gapless Dirac fermions. In the new basis, the matrices in the Dirac equation are

$$\gamma^1=\frac{1}{\sqrt{6}}(-2\mu^3+\mu^1-\mu^2)$$
$$\gamma^2=\frac{1}{\sqrt{2}}(\mu^1+\mu^2)$$
$$\gamma^0=\frac{1}{\sqrt{3}}(-\mu^3-\mu^1+\mu^2) \tag{8}$$

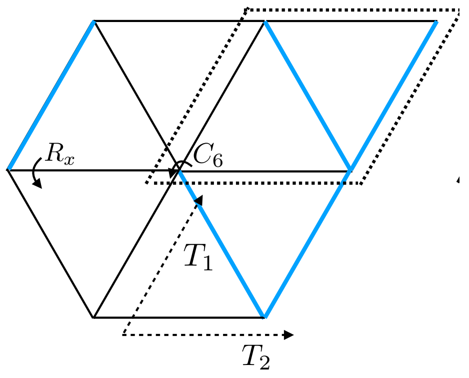

Supplementary Figure 1: The mean-field choice on triangular lattice to realize the staggered flux configuration. Blue(Black) bonds has ∓1 hopping amplitudes, respectively.

For later purposes, the charge conjugate operation is given here as

$$\psi \to W_c\psi^* = \frac{1}{\sqrt{3}}(-iI_{4\times 4} - \mu^3 + \mu^1)\psi^* \tag{9}$$

The PSG for all the symmetry operations transform as the following:

$$\psi \xrightarrow{T_1} -i\tau^2\psi \qquad \psi \xrightarrow{T_2} i\tau^3\psi \qquad \psi \xrightarrow{\mathcal{T}} i\sigma^2\mu^2\tau^2\psi(-k) \tag{10}$$

$$\psi(k_1,k_2) \xrightarrow{C_6} i\sigma^2 W_{C_6}\psi^\dagger(-\frac{k_2}{2}, 2k_1 - k_2) \qquad \psi(k_1,k_2) \xrightarrow{R} i\sigma^y W_R\psi^\dagger(k_1 - \frac{k_2}{2}, -k_2) \tag{11}$$

where

$$W_{C_6} = e^{-i\gamma^3\frac{\pi}{6}} W_c exp[i\frac{\pi}{3}\tau^C] \quad \tau^C = \frac{1}{\sqrt{3}}(\tau^1 + \tau^2 + \tau^3)$$
$$W_R = \frac{(\gamma^1 - \sqrt{3}\gamma^2)}{2} W_c \frac{\tau^3 - \tau^1}{\sqrt{2}} \tag{12}$$

There are the following defining relations of the symmetry group which gives constraints on Berry phase

$$T_2^{-1}T_1^{-1}T_2T_1 = e \quad [\mathcal{T}, T_{1/2}] = 0$$
$$T_1RT_1 = T_2R \quad T_1C_6 = C_6T_2 \quad T_2C_6 = C_6T_1^{-1}T_2$$
$$R^2 = 1 \quad \mathcal{T}R = R\mathcal{T} \quad C_6RC_6 = R$$
$$\mathcal{T}C_6 = C_6\mathcal{T} \quad C_6^6 = 1 \quad \mathcal{T}^2 = 1 \tag{13}$$

Applying the above algebraic relation to monopole transformations, we find the following constraints on the Berry phase for translation $T_{1/2} : \theta_{1/2}$ and reflection $R : \theta_R$ aswe could fix other phase factors to be

$$\theta_R = 0, \pi \quad \theta_1 = \pm\frac{2\pi}{3}p, 0 \quad \theta_2 = 2\theta_1. \tag{14}$$

and numerically we find $\theta_1 = \frac{2\pi}{3}, \theta_R = 0$.

D. Kagome

On kagome lattice, similar to triangular case, Hermele et al calculated the kagome DSL with staggered flux mean-field ansatz, with three gamma matrices as $\gamma_\nu = (\mu^3, \mu^2, -\mu^!)$, and we have for the PSG of Dirac fermions as

$$T_1 : \psi \to (i\tau^2)\psi \quad T_2 : \psi \to (i\tau^3)\psi \quad R_y : \psi \to (i\mu^1)exp(\frac{i\pi}{2}\tau_{ry})\psi$$
$$C_6 : \psi \to exp(\frac{i\pi}{3}\mu^3)exp(\frac{2\pi i}{3}\tau_R)\psi \quad \mathcal{T} : \psi \to (i\sigma^2)(i\mu^2)(-i\tau^2)\psi. \tag{15}$$

where

$$\tau_{ry} = \frac{-1}{\sqrt{2}}(\tau^1 + \tau^3) \quad \tau_R = \frac{1}{\sqrt{3}}(\tau^1 + \tau^2 - \tau^3). \tag{16}$$

The algebraic relations used to fix translation and rotation Berry phase read:

$$\mathcal{T}^2 = R_y^2 = (C_6)^6 = I \quad \mathcal{T}G = G\mathcal{T}, G = T_1, T_2, R_y, C_6$$
$$(C_6)^6 = 1 \quad T_1T_2 = T_2T_1 \quad C_6T_1 = T_2C_6 \quad T_1C_6T_2 = T_2C_6 \quad T_2R_yT_2 = T_1R_y \tag{17}$$

which fix the Berry phase for translation to be zero $\theta_1 = \theta_2 = 0$ (notice this is the result of relations like $C_6T_1 = T_2C_6$, which means rotation selects discrete values for momenta); the phase for rotation $\theta_C = n\pi/3$ while we numerically find $\theta_C = 2\pi/3$.

Supplementary Note 2. SIGN AMBIGUITY OF BERRY PHASE

Here we remark on the sign ambiguity of Berry phase. As stated in the main text, there is a Z_2 element in both $SO(6)$,i.e., its center and $U(1)_{top}$, i.e., -1 that act identically on all physical operators, i.e., trivially on fermion bilinears and giving a minus sign for all 6 monopoles. Berry phase, by definition, is the element in $U(1)_{top}$ under certain symmetry transformation that is embedded into the emergent symmetry group $SO(6) \times U(1)_{top}/\mathbb{Z}_2$; therefore whether the $\mathbb{Z}_2$ operation belongs to $SO(6)$ or $U(1)_{top}$,i.e., Berry phase, is arbitrary and up to one's choice of convention. In other words, certain PSG $\psi \to W\psi$ where W is an $SU(4)$ matrix can be changed to an equally good PSG $\psi \to iW\psi$ since iW is also an $SU(4)$ matrix. While this additional i factor has no effect on fermion bilinears, it corresponds to the center in $SO(6)$ which changes sign for all monopoles. The physical symmetry operation should stay invariant regardless this change in PSG meaning the Berry phase should change by π to compensate the center in $SO(6)$.

In our numerics and Berry phase analysis by algebraic relations, we define the $SO(6)$ element by the transformation of 6 fermion bilinears $\bar{\psi}\tau^{1/2/3}\psi, \bar{\psi}\sigma^{1/2/3}\psi$ to eliminate this sign ambiguity. This is possible because actually all physical symmetries act within the $SO(3)_{valley} \times SO(3)_{spin}$ subgroup of the $SO(6)$, and the three valley(spin) hall masses $\bar{\psi}\tau^{1/2/3}\psi$,$\bar{\psi}\sigma^{1/2/3}\psi$, which are adjoint representation for $SO(3)_{valley/spin}$, respectively, happen to constitute also the vector representation of $SO(3)_{valley} \times SO(3)_{spin}$. Hence they can be used as the reference frame with regard to which the Berry phase is defined.

Supplementary Note 3. NUMERICS ON KAGOMÈ LATTICE

On the kagomè lattice, we numerically find $\langle\psi|G_{C_6} \cdot C_6|\psi\rangle = e^{i2\pi/3}$, hence the lattice angular momentum of spin triplet monopole is $2\pi/3$. Specifically, we have a $L \times L$ kagomè lattice on a torus, and consider the parton mean-field ansatz of Dirac spin liquid with uniformly spreading 2π flux on the kagomè lattice (see Supplementary Fig. 2),

$$\begin{aligned} H = \sum & e^{iA_1(\mathbf{r})} f_a^\dagger(\mathbf{r}) f_b(\mathbf{r}) + e^{iA_2(\mathbf{r})} f_a^\dagger(\mathbf{r}) f_c(\mathbf{r}) \\ & + e^{iA_3(\mathbf{r})} f_b^\dagger(\mathbf{r}) f_c(\mathbf{r}) + e^{iA_4(\mathbf{r})} f_b^\dagger(\mathbf{r}) f_c(\mathbf{r}+\mathbf{e}_x-\mathbf{e}_y) \\ & + e^{iA_5(\mathbf{r})} f_b^\dagger(\mathbf{r}) f_a(\mathbf{r}+\mathbf{e}_x) + e^{iA_6(\mathbf{r})} f_c^\dagger(\mathbf{r}) f_a(\mathbf{r}+\mathbf{e}_y) + h.c. \end{aligned} \tag{18}$$

where the gauge fields $A(\mathbf{r} = (x, y))$, $x, y = 0, 1, \cdots, L-1$, are

$$\begin{aligned} & A_1(\mathbf{r}) = \frac{\pi}{2L^2}, \quad A_2(\mathbf{r}) = x\pi + \frac{x\pi}{L^2} + \frac{\pi}{2L^2} \\ & A_3(\mathbf{r}) = x\pi + \frac{(x+1/4)\pi}{L^2}, \\ & A_4(\mathbf{r}) = \begin{cases} -\dfrac{(x+3/4)\pi}{L^2}, & x \neq L-1 \\ \dfrac{\pi}{4L^2} - \dfrac{2y\pi}{L}, & x = L-1 \end{cases} \\ & A_5(\mathbf{r}) = \begin{cases} \dfrac{\pi}{2L^2}, & x \neq L-1 \\ \dfrac{\pi}{2L^2} - \dfrac{2y\pi}{L}, & x = L-1 \end{cases} \\ & A_6(\mathbf{r}) = \frac{x\pi}{L^2} + \frac{\pi}{2L^2}. \end{aligned} \tag{19}$$

We further diagonalize the single-particle Hamiltonian, and construct the monopole state $|\psi\rangle$ by filling the Dirac sea as well as two spin-up zero modes. Finally we numerically obtain the lattice angular momentum, $\langle\psi|G_{C_6} \cdot C_6|\psi\rangle = e^{i2\pi/3}$. Note, in this particular case we are able to retain symmetry in the state with a single monopole insertion.

Supplementary Note 4. SYMMETRY-ALLOWED HIGHER-ORDER MONOPOLES ON KAGOME & TRIANGULAR LATTICES

For Kagome lattices, we consider the transformations of 4π monopoles and look for a symmetry-allowed double monopole. Under 4π magnetic flux, each Dirac fermion bears 2 zero modes, carrying Lorenz spin$-1/2$, denoted by $f_{k,s,\pm}$ where $\pm$ associate to Lorentz index.

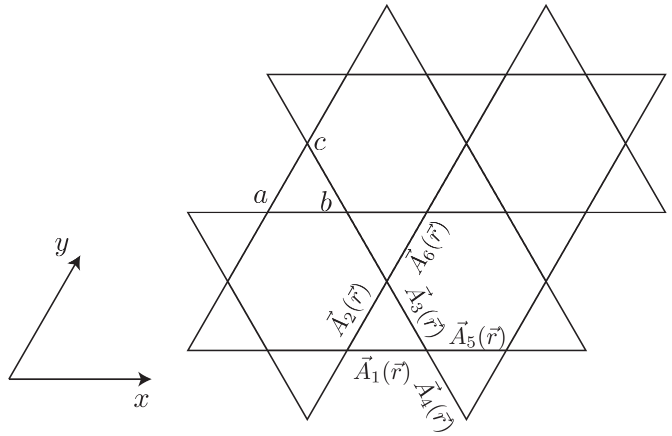

Supplementary Figure 2: Gauge field ansatz for Dirac spin liquid with 2π monopole flux on the kagomè lattice.

On kagome lattice, three gamma matrices are $\gamma_\nu = (\mu^3, \mu^2, -\mu^1)$ (acting in Lorenz index space), and we have for the PSG of Dirac fermions as

$$T_1 : \psi \to (i\tau^2)\psi \quad T_2 : \psi \to (i\tau^3)\psi \quad R_y : \psi \to (i\mu^1)exp(\frac{i\pi}{2}\tau_{ry})\psi$$
$$C_6 : \psi \to exp(\frac{i\pi}{3}\mu^3)exp(\frac{2\pi i}{3}\tau_R)\psi \quad \mathcal{T} : \psi \to (i\sigma^2)(i\mu^2)(-i\tau^2)\psi. \tag{20}$$

where

$$\tau_{ry} = \frac{-1}{\sqrt{2}}(\tau^1 + \tau^3) \quad \tau_R = \frac{1}{\sqrt{3}}(\tau^1 + \tau^2 - \tau^3). \tag{21}$$

where τ, σ act in valley (k) and physical spin (s) spaces and zero modes transform as Dirac fermions.

Leading 4π monopole consists of filling Dirac sea $|4\pi\rangle$ and 4 out of 8 zero modes, giving 70 4π monopoles. Consider following three such monopoles $\tilde{\Phi}_i^\dagger$ made of

$$\tilde{\Phi}_i^\dagger = [\sum_p p f^\dagger_{k,s,p}(\epsilon\tau^i)^{k,k'}\epsilon^{s,s'}f^\dagger_{k',s',-p}]^2|4\pi\rangle, \tag{22}$$

which are spin σ singlets, and $p = \pm$ denotes the lorentz index. These constitute Lorentz singlets and don't vanish even though zero modes are fermionic. Berry phases are twice for $\tilde{\Phi}_i$ as for elementary Φ_i and $SO(3)_{valley}$ part of the symmetry permutes the indices of τ's (i.e., i in $\tilde{\Phi}_i$) at most.

The above leads to $\tilde{\Phi}_i^\dagger \sim [\mathcal{V}_i^\dagger]^2$ under physical symmetries and hence the symmetry-allowed 2-fold monopole terms read

$$\Delta\mathcal{L}_{kagome} = e^{i2\pi/3}\tilde{\Phi}_1^\dagger + \tilde{\Phi}_2^\dagger + e^{-i2\pi/3}\tilde{\Phi}_3^\dagger + h.c., \tag{23}$$

where scaling dimension ≈ 2.5.

The another symmetry allowed composite monopole on Kagome reads

$$\Delta\mathcal{L}^1_{\text{kagome}} = M_{01}(\Phi_1 e^{i\frac{2\pi}{3}}) + M_{02}(\Phi_2) + M_{03}(\Phi_3 e^{-i\frac{2\pi}{3}}) + \text{h.c.} \tag{24}$$

which amounts to an excited *lorentz singlet* 2π monopole. The leading-order operator in this kind results from exciting one landau level $n = -1$ mode to $n = 1$ and has dimension $\Delta_0 + 2\sqrt{2} \approx 3.8$ which is irrelevant. In the operator product expansion of the above term, the monopole carries vanishing lorentz spin and hence the coefficient of excited monopole from exciting $n = 0$ to $n = 1$ vanishes. We could also rule out the case with a lower dimension where a zero mode is excited to $n = 1$ Landau level since then, the monopole will inevitably carry lorentz spin 1 and won't be invariant under the lorentz group part $Exp(i\mu^3\pi/3)$ for C_6 and $i\mu^1$ for R in eq(20) simultaneously.

For triangular lattice, the leading-order symmetry-allowed is 3−fold monopoles and the zero modes carry lorentz spin 1. Similarly, we construct lorentz singlet out of lorentz spin 1 zero modes and they transform formally as

$\Phi_1\Phi_2\Phi_3$, i.e., the corresponding 6π monopole consists of

$$\tilde{\Phi}_{6\pi} = \prod_{i=1,2,3} [f^\dagger_{k,s,1}(\epsilon\tau^i)^{k,k'}\epsilon^{s,s'}f^\dagger_{k',s',-1} - f^\dagger_{k,s,0}(\epsilon\tau^i)^{k,k'}\epsilon^{s,s'}f^\dagger_{k',s',0} + f^\dagger_{k,s,-1}(\epsilon\tau^i)^{k,k'}\epsilon^{s,s'}f^\dagger_{k',s',1}]|6\pi\rangle, \tag{25}$$

where $\pm 1, 0$ labels lorentz spin and each factor labeled by valley index $i = 1, 2, 3$ creates two zero modes that carries total 0 lorentz spin (i.e., the lorentz index part amounts to $|1,-1\rangle - |0,0\rangle + |-1,1\rangle$ which is a singlet) and transform as the associated elementary monopole Φ_i for the $SO(6)$ flavor part. Hence we've got a lorentz singlet symmetry-allowed monopole on triangular lattices $\tilde{\Phi}_{6\pi} + \tilde{\Phi}^\dagger_{6\pi}$, dimension likely irrelevant.

Supplementary Note 5. UNCONVENTIONAL ORDERS PROXIMATE TO DSL

In this appendix we discuss the unconventional orders that could descend from DSL theory. We consider the "mixed" fermion mass $\pm\bar{\psi}\sigma^i\tau^j\psi$ on different lattices and the quantum spin hall mass on bipartite lattices. The general strategy relating mass and monopole proliferation to symmetry-breaking orders is to find the microscopic spin operators (as simple as possible for practical purposes) that transform in the same way as the mass and monopoles (they do not have to be identical). The ordered states should have orders given by all of the microscopic operators.

With a "mixed" mass $\pm\bar{\psi}\sigma^i\tau^j\psi$, monopole $\mathcal{V}_j \mp i\mathcal{S}_i$ will proliferate depending on the sign of the mass. The conventional wisdom is that excluding square lattice case, mass and $\mathcal{S}_i$ is time-reversal odd hence corresponding to spinful operators, simplest of which is a single spin operator (which we will show later does not suffice for triangular/square lattices); while $\mathcal{V}_j$ is time-reversal even and does not carry spins, corresponding to valence bond operators $S_i \cdot S_j$; hence this scenario leads to a mixture of spin and valence bond order. One remarkable feature is that mass and monopoles all preserve spin rotation along σ^i direction, leading to collinear spin orders along this direction, if any.

Now we illustrate some simple or relatively symmetric order patterns resulting from $\pm\bar{\psi}\sigma^i\tau^j\psi$ and monopoles.

On triangular lattice, first consider a typical such mass, $M_{33} = \bar{\psi}\sigma^3\tau^3\psi$ which favors $\mathcal{V}_3 + i\mathcal{S}_3$. We consider the case where $\langle\mathcal{V}_3 + i\mathcal{S}_3\rangle = 1$, plotted as Supplementary Fig 3(1). The spins should order along z direction. If we write down a trial association

$$\langle\mathcal{S}_3\rangle \sim i\sum_r S^z_r e^{iQ\cdot r} \tag{26}$$

where $Q = (\frac{\pi}{3}, \frac{2\pi}{3})$ is $\mathcal{S}_3$'s momentum, we check that this operator has the same symmetry group as $\mathcal{S}_3$, i.e., aside from translations, $\mathcal{S}_3$ preserves reflection along e_2 direction (C_6^2R in main text notation) marked in Supplementary fig 3(1).

For $\mathcal{V}_3$, similarly we construct such correspondence

$$\langle\mathcal{V}_3\rangle + A\langle\mathcal{V}_3\rangle^2 + B\langle\mathcal{V}_3\rangle^3 + \cdots \sim (\sum_r)' P_{vbs}(r) \tag{27}$$

where $P_{vbs}(r)$ denotes certain combinations of valence bond operator $S_{r1} \cdot S_{r2} + \frac{1}{4}$ inside the enlarged unit cell at r defined by the momentum of $\mathcal{V}_3 : (\frac{\pi}{3}, \frac{2\pi}{3})$, and $(\sum_r)'$ sums over new Bravais lattice with the enlarged unit cell. The difference here is the higher order terms of $\mathcal{V}_3$ on LHS of eq (27) which generically should appear (not forbidden by any quantum number considerations) [1] and hence the VBS patterns do not have to be at the same momenta of $\mathcal{V}_3$ – so long as they have the same enlarged unit cell. Under reflection equivalent to C_6^2R, $\mathcal{V}_3$ stays invariant, which means an additional constraint on $P_{vbs}(r)$ to be reflection invariant.

The association of M_{33} to spins is a bit involved since the mass has definite momentum but is odd under reflection (Supplementary table 1), which leaves certain sites invariant, rendering it inconsistent to relate to a single spin operator. We find the simplest microscopic operators corresponding to M_{33} contain three spin terms describing some "bond-centered" spin moments,

$$M_{33} \sim \sum_r e^{iQ_M\cdot r}[S_{r+e1} \times (S_r \times S_{r+e2}) - S_{r+e3} \times (S_r \times S_{r+e2})] \tag{28}$$

[1] This can not happen for the case of spin order because they carry nonvanishing spin quantum numbers.

where $e_{1/2/3}$ are three unit lattice vectors shown in Supplementary fig 3(1), Q_M is the momentum of M_{33}. This three spin terms are reflection odd while invariant under $(C_6)^3$ as the mass.

Equipped with the above 3 types of order parameters eqs (26)(27)(28), we finally come at the order pattern drawn in Supplementary fig 3(1) that contains nonzero components of all 3 order parameters. The momenta of $M_{33}, \mathcal{S}_3, \mathcal{V}_3$ are $(\pi, 0), (-\frac{2\pi}{3}, \frac{2\pi}{3}), (\frac{\pi}{3}, \frac{2\pi}{3})$ in the convention of main text, respectively, which gives the new lattice vectors $R_{1/2}$ of the order pattern as marked arrows in Supplementary Fig 3 (1). We remark that under inversion M_{33} stays invariant, $\mathcal{V}_3 \to \mathcal{V}_3^\dagger, \mathcal{S}_3 \to -\mathcal{S}_3^\dagger$; hence under the particular expectation value $\langle \mathcal{V}_3 + i\mathcal{S}_3 \rangle = 1$, inversion is also preserved. We see the ordered pattern has 6-site unit cell

Another interesting case is for the mass $M_{31} + M_{32} - M_{33}$ which is C_6 invariant (Supplementary table 1), drawn in Supplementary Fig 3(2). The corresponding monopole $\Phi_{tri} = \frac{1}{\sqrt{3}}(\mathcal{V}_1 + \mathcal{V}_2 - \mathcal{V}_3) + i\mathcal{S}_3$ condenses. Analogously one finds microscopic operators consistent with these low-energy objects and constructs exemplar ordered pattern. Note Φ_{tri} goes to $\Phi_{tri}^\dagger$ under C_6. Hence if $\langle \Phi_{tri} \rangle = 1$, the pattern should preserve C_6. However, now the pattern has larger unit cell with 12 sites since the new smallest lattice vectors that render both mass and monopole invariant $R_{1/2}$ in Supplementary Fig 3(2) is larger.

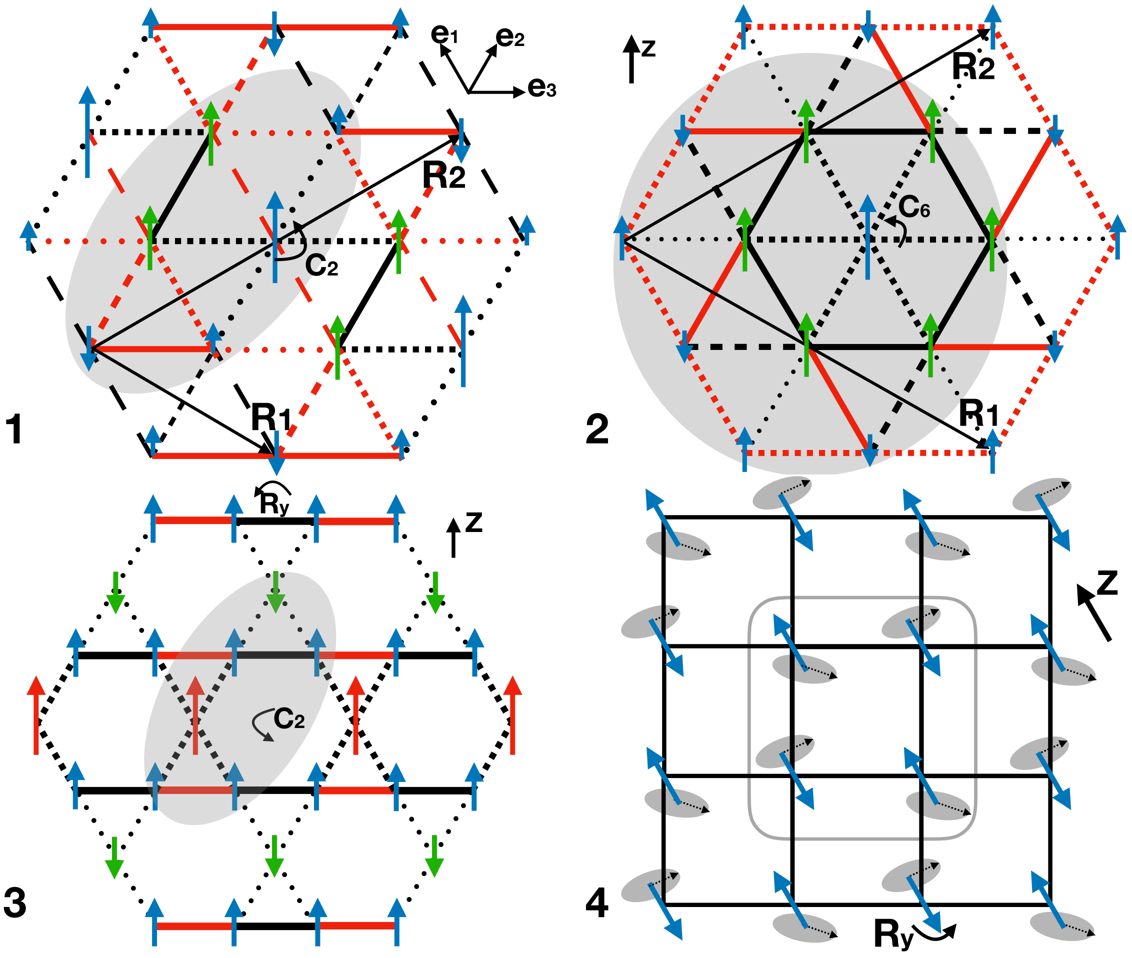

Supplementary Figure 3: Spin-valence-bond mixed order on triangular, kagomè and square lattices. Blue/green arrows indicate spins with the lengths schematically showing the spin component along z direction. Bonds in different color/patterns are associated with different valence bond weights. Plotted are schematic order patterns dictated by the symmetry group of mass and monopoles. Marked are the enlarged unit cell and other symmetries preserved by order parameters. 1 results from M_{33} and preserves reflection along direction marked by black curved arrow, inversion, translations along $R_{1/2}$ with 6-site unit cell. 2 results from $M_{31} + M_{32} - M_{33}$ and preserves C_6, translations along $R_{1/2}$ with 12-site unit cell. 3 results from M_{32} and preserves $T_1, [T_2]^2, R_y$ and inversion, doubling the original unit cell on kagomè lattice. 4 results from M_{33} on square lattice with 4-site unit cell and collinear spin order. The gray ovals denote spins fluctuating coherently in the xy plane giving rise to orders $\mathbf{S}_r \times \mathbf{S}_{r+e2}$.

For kagomè lattice, similarly one considers the mass M_{32} which favors $\mathcal{V}_2 + i\mathcal{S}_3$. Here we can safely associate both M_{32} (reflection even) and $\mathcal{S}_3$ to some simple spin moments

$$M_{32} \sim \sum_r e^{iQ_M \cdot r} P_{Sz}(r)$$
$$\langle \mathcal{S}_3 \rangle \sim \sum_r e^{iQ_s \cdot r} G_{Sz}(r) \tag{29}$$

where $P_{Sz}(r), G_{Sz}(r)$ are certain combination of spin S_z moments inside one unit cell located at r, and $Q_{M/s}$ are momenta of the mass and $\mathcal{S}_3$. The VBS order parameter associated with $\mathcal{V}_2$ is identical to eq (27) with the enlarged unit cell (doubled along direction of T_2 translation) consistent with momentum of $\mathcal{V}_2$. And if $\langle \mathcal{V}_2 + i\mathcal{S}_3 \rangle = 1$ (purely real), this further preserves $[C_6]^3, R_y$ in addition to $T_1, [T_2]^2$. Drawn in Supplementary Fig 3(3) is a schematic pattern that is consistent with these symmetries.

For square lattice, the mixed mass M_{i2} belong to the $SO(5)$ vectors discussed in main text. The other mixed mass, e.g., M_{33} is even under time reversal and can be associated to

$$(M_{13}, M_{23}, M_{33}) \sim \sum_r (-1)^{r1} S_r \times S_{r+e2}. \tag{30}$$

where $e_{1/2}, r_{1/2}$ is the unit lattice vector/site coordinates along horizontal/vertical direction, respectively.

$-M_{33}$ will proliferate $\mathcal{V}_3 + i\mathcal{S}_3$. A simple case is $\langle \mathcal{V}_3 + i\mathcal{S}_3 \rangle = i$, which means Neel order along σ^3 direction associated with $Re[\mathcal{S}_3]$ and the order for M_{33} expressed in eq (30)[2]. So spins order anti-ferromagnetically along z direction while also fluctuates in the xy plane coherently. This preserves $[T_1]^2, [T_2]^2, R_y$ with 4-site unit cell shown schematically in Supplementary fig 3(4).

In the case for quantum spin hall mass, $\bar{\psi}\sigma^i\psi$ and monopole $\mathcal{S}_j + i\mathcal{S}_k$ results in spin order that *fully* breaks $SO(3)_{spin}$. For quantum spin hall mass on square lattice, we use following spin operators

$$(M_{10}, M_{20}, M_{30}) \sim \mathbf{S}_{spin-hall} \equiv \mathbf{S}_0 \times \sum_r (-1)^{r_1+r_2}[S_r \times S_{r+e1+2e2} - S_r \times S_{r-2e2+e1} + S_r \times S_{r+2e1+e2} - S_r \times S_{r+2e1-e2}). \tag{31}$$

and $\mathbf{S}_0 = \frac{1}{N}\sum_r \mathbf{S}_r$ is the uniform component of lattice spin moments. This is odd under translations, reflections, rotations and time-reversal, and consistent with symmetries of quantum spin hall mass.

In the staggered flux phase, the monopoles do not have definite time reversal quantum numbers. The Neel order $\mathbf{S}_{neel}$ from M_{i2} in the main text is related to $Re[\mathcal{S}_i]$. The imaginary part of $\mathcal{S}_i$ transforms as

$$(Im[\mathcal{S}_1], Im[\mathcal{S}_2], Im[\mathcal{S}_3]) \sim \mathbf{S}_{spin-hall} \times \mathbf{S}_{neel}. \tag{32}$$

The real part of $\mathcal{S}_i$'s has the symmetry of Neel order $\mathbf{S}_{neel}$, and Hence the ordered state under the quantum spin hall mass is captured by 2 order parameters, $\mathbf{S}_{neel}, \mathbf{S}_{spin-hall}$ that aligns along the $Re[\langle \mathcal{S} \rangle], M_{0i}$ direction, respectively, orthogonal to each other. The co-existence of the neel order and products like $S_i \times S_j$ shows the quantum nature of the ordered state – the spins fluctuate in a coherent way while on average they order anti-ferromagnetically.

On honeycomb lattice, we have

$$(M_{10}, M_{20}, M_{30}) \sim \mathbf{S}_{spin-hall} = \sum_r S_r \times S_{r+\epsilon^r e1} + S_r \times S_{r+\epsilon^r e2} + S_r \times S_{r-\epsilon^r e1-\epsilon^r e2} \tag{33}$$

where $\epsilon^r = \pm 1$ depending on which sublattice site r belong to and $e_{1/2}$ is the unit vector along translation $T_{1/2}$ with $2\pi/3$ angle between them. This is rotation invariant with $(0,0)$ momentum and reflection odd. Similar to square lattice, the imaginary part of $\mathcal{S}_i$ behave just like the Neel order parameter S_{neel} along i direction while

$$(Re[\mathcal{S}_1], Re[\mathcal{S}_2], Re[\mathcal{S}_3]) \sim \mathbf{S}_{neel} \times \mathbf{S}_{spin-hall}. \tag{34}$$

Note the spin order is collinear, since both the mass and $\mathcal{S}_i$'s are even under $\mathcal{T}C_6$. The resulting order is an inherently quantum "Neel" order, with order described by $\mathbf{S}_{neel}, \mathbf{S}_{spin-hall}$, identical to the chiral antiferromagnetic phase found in ref [2, 3].

[2] $Im[\mathcal{V}_3]$ transforms as $(M_{13}, M_{23}, M_{33}) \cdot (Re[\mathcal{S}_1], Re[\mathcal{S}_2], Re[\mathcal{S}_3])^T$ so it is enough to condense both

Lattice	Bilinears	T_1	T_2	$Reflection$	$Rotation$	$\mathcal{T}$
square	M_{00}	$+$	$+$	$-$	$+$	$-$
	M_{i0}	$-$	$-$	$-$	$-$	$-$
	M_{01}	$-$	$+$	$+$	M_{03}	$+$
	M_{02}	$+$	$+$	$+$	$-M_{02}$	$-$
	M_{03}	$+$	$-$	$-$	$-M_{01}$	$+$
	M_{i1}	$+$	$-$	$+$	$-M_{i3}$	$+$
	M_{i2}	$-$	$-$	$+$	M_{i2}	$-$
	M_{i3}	$-$	$+$	$-$	M_{i1}	$+$
honey-comb	M_{00}	$+$	$+$	$-$	$+$	$-$
	M_{i0}	$+$	$+$	$-$	$+$	$+$
	M_{01}	$\cos(\frac{2\pi}{3})M_{01}+\sin(\frac{2\pi}{3})M_{02}$		$+$	$\cos(\frac{2\pi}{3})M_{01}+\sin(\frac{2\pi}{3})M_{02}$	$+$
	M_{02}	$\cos(\frac{2\pi}{3})M_{02}-\sin(\frac{2\pi}{3})M_{01}$		$-$	$-\cos(\frac{2\pi}{3})M_{02}+\sin(\frac{2\pi}{3})M_{01}$	$+$
	M_{03}	$+$	$+$	$+$	$-$	$+$
	M_{i1}	$\cos(\frac{2\pi}{3})M_{i1}+\sin(\frac{2\pi}{3})M_{i2}$		$+$	$\cos(\frac{2\pi}{3})M_{i1}+\sin(\frac{2\pi}{3})M_{i2}$	$-$
	M_{i2}	$\cos(\frac{2\pi}{3})M_{i2}-\sin(\frac{2\pi}{3})M_{i1}$		$-$	$-\cos(\frac{2\pi}{3})M_{i2}+\sin(\frac{2\pi}{3})M_{i1}$	$-$
	M_{i3}	$+$	$+$	$+$	$-$	$-$
triangle	M_{00}	$+$	$+$	$-$	$+$	$-$
	M_{i0}	$+$	$+$	$+$	$-$	$+$
	M_{01}	$-$	$-$	$-M_{03}$	$-M_{02}$	$+$
	M_{02}	$+$	$-$	M_{02}	M_{03}	$+$
	M_{03}	$-$	$+$	$-M_{01}$	M_{01}	$+$
	M_{i1}	$-$	$-$	M_{i3}	M_{i2}	$-$
	M_{i2}	$+$	$-$	$-M_{i2}$	$-M_{i3}$	$-$
	M_{i3}	$-$	$+$	M_{i1}	$-M_{i1}$	$-$
kagome	M_{00}	$+$	$+$	$-$	$+$	$-$
	M_{i0}	$+$	$+$	$-$	$+$	$+$
	M_{01}	$-$	$-$	$-M_{03}$	M_{02}	$+$
	M_{02}	$+$	$-$	M_{02}	$-M_{03}$	$+$
	M_{03}	$-$	$+$	$-M_{01}$	$-M_{01}$	$+$
	M_{i1}	$-$	$-$	$-M_{i3}$	M_{i2}	$-$
	M_{i2}	$+$	$-$	M_{i2}	$-M_{i3}$	$-$
	M_{i3}	$-$	$+$	$-M_{i1}$	$-M_{i1}$	$-$

Supplementary Table 1: The transformation of fermion bilinears $M_{ij} \equiv \overline{\psi}\sigma^i\tau^j\psi$ on four types of lattices. $T_{1/2}$ denotes translation along two lattice vectors defined on each lattice type in Supplementary fig **??**(staggered flux mean field on square lattice), reflection denotes reflection perpendicular to the horizontal bond for square/honeycomb/triangular and vertical direction for kagomè lattices, rotation denotes 4−fold rotation around site for square, 6−fold rotation for honeycomb/kagomè/triangular lattices.

[1] Xue-Yang Song, Yin-Chen He, Ashvin Vishwanath, and Chong Wang, "From spinon band topology to the symmetry quantum numbers of monopoles in Dirac spin liquids," arXiv e-prints , arXiv:1811.11182 (2018), arXiv:1811.11182 [cond-mat.str-el].

[2] Fa Wang, "Schwinger boson mean field theories of spin liquid states on a honeycomb lattice: Projective symmetry group analysis and critical field theory," Phys. Rev. B **82**, 024419 (2010).

[3] Yuan-Ming Lu and Ying Ran, "$\mathcal{Z}_2$ spin liquid and chiral antiferromagnetic phase in the hubbard model on a honeycomb lattice," Phys. Rev. B **84**, 024420 (2011).

$M_{33}, Re[\mathcal{S}_3]$.
